# Association of Head Injury, Neck Injury or Acoustic Trauma on Phenotype of Ménière’s Disease

**DOI:** 10.3390/audiolres14010019

**Published:** 2024-02-17

**Authors:** Ilmari Pyykkö, Vinay, Artur Vetkas, Jing Zou, Vinaya Manchaiah

**Affiliations:** 1 Hearing and Balance Research Unit, Field of Otolaryngology, Tampere University, 33100 Tampere, Finland; ilmari.pyykko@tuni.fi (I.P.); jing.zou@tuni.fi (J.Z.); 2 Department of Neuromedicine and Movement Sciences, Norwegian University of Science and Technology, 7491 Trondheim, Norway; 3 Department of Otolaryngology-Head and Neck Surgery, University of Colorado School of Medicine, Aurora, CO 80045, USA; vinaya.manchaiah@cuanschutz.edu; 4 Faculty of Medicine, University of Tartu, 50090 Tartu, Estonia; 5 Department of Otolaryngology-Head and Neck Surgery, Center for Otolaryngology-Head & Neck Surgery of the Chinese PLA, Changhai Hospital, Second Military Medical University, Shanghai 201823, China; 6 UCHealth Hearing and Balance, University of Colorado Hospital, Aurora, CO 80045, USA; 7 Virtual Hearing Lab., Collaborative Initiative between University of Colorado School of Medicine and University of Pretoria, Aurora, CO 80045, USA; 8 Department of Speech-Language Pathology and Audiology, University of Pretoria, Pretoria 0001, South Africa; 9 Department of Speech and Hearing, School of Allied Health Sciences, Manipal University, Manipal 576104, Karnataka, India

**Keywords:** Ménière’s disease, complaint profile, head injury, loss of consciousness, vertigo with neck injury, vestibular drop attack, quality of life

## Abstract

The aim of the present study was to investigate adverse effects of head injury, neck trauma, and chronic noise exposure on the complaint profile in people with Ménière’s disease (MD). The study used a retrospective design. Register data of 912 patients with MD from the Finnish Ménière Federation database were studied. The data comprised case histories of traumatic brain injury (TBI), neck trauma and occupational noise exposure, MD specific complaints, impact related questions, and the E-Qol health-related quality of life instrument. TBI was classified based on mild, moderate, and severe categories of transient loss of consciousness (TLoC). The mean age of the participants was 60.2 years, the mean duration of the disease was 12.6 years, and 78.7% were females. Logistic regression analysis, linear correlation, and pairwise comparisons were used in evaluating the associations. 19.2% of the participants with MD had a history of TBI. The phenotype of participants with TBI was associated with frequent vestibular drop attacks (VDA), presyncope, headache-associated vertigo, and a reduction in the E-QoL. Logistic regression analysis explained the variability of mild TBI in 6.8%. A history of neck trauma was present in 10.8% of the participants. Neck trauma associated with vertigo (NTwV) was seen in 47 and not associated with vertigo in 52 participants. The phenotype of NTwV was associated with balance problems, VDA, physical strain-induced vertigo, and hyperacusia. Logistic regression analysis explained 8.7% of the variability of the complaint profile. Occupational noise exposure was recorded in 25.4% of the participants and correlated with the greater impact of tinnitus, hyperacusis, and hearing loss. Neither the frequency, duration, or severity of vertigo or nausea were significantly different between the baseline group and the TBI, NTwV, or noise-exposure groups. The results indicate that TBI and NTwV are common among MD patients and may cause a confounder effect.

## 1. Introduction

According to the World Health Organisation (WHO), two out of three Europeans of retirement age will have at least two chronic medical conditions [1]. Meniére’s disease (MD) is one such chronic disease with a variable phenotype and a major impact on quality of life [2]. As the etiology of MD is not well understood, a significant number of patients are questioning whether their symptoms originate from diverse events such as infections, head or neck injury, and noise exposure, among others. So far, the causal association of complaints in MD after traumatic brain injury (TBI) or blast exposure are poorly understood, although some authors associate some of these events with vestibular dysfunction [3]. TBI has been associated with benign paroxysmal positional vertigo (BPPV), complaints of dizziness, and balance problems [4,5].

Traumatic brain injury (TBI) is an enormous public-health concern, with up to 20% of the population experiencing it at some point in their lives [6]. The incidence of TBI in Finland is 101 for 100,000 inhabitants, and the most common external causes are related to falls [7]. Among the most common complaints after mild TBI are headaches, dizziness, fatigue, and cognitive difficulties, with about 13% unable to continue to work [5,8]. The variability in incidence rate may depend on definitions of mild TBI and time elapsed since the injury. The incidence of dizziness with even mild TBI is common and ranges from 17% to 72% [8,9]. In one of the few long-term studies on untreated patients with mild TBI, Berman and Frederickson [9] showed vertigo persisting in 59% of the patients after five years of recovery. Lee et al. [4] reported otolith problems and non-specific dizziness in a majority of their TBI patients, and their rehabilitation was relatively unsuccessful. Misale et al. [8] measured saccular and utricular function with cervical and ocular vestibular-evoked myogenic potentials (cVEMP and oVEMP), respectively, and their results showed abnormalities in both otolith organs after TBI. In the animal model, head trauma may detach the otoconias, leading to long-term changes in the utricle [3]. This mechanism has also been suggested for the etiology of benign positional vertigo after TBI in humans [10,11].

TBI can induce endolymphatic hydrops in experimental animals, and such connection has been also suggested in humans [12]. The reported incidence of hearing loss shortly after mild TBI ranges from 7% to 50% [13] and is higher in severe TBI with temporal bone fractures [14]. Moreover, hearing loss occurs at high frequencies and is usually not fluctuating [5].

The correlation between dizziness and neck injury is supported by an associated frequency of inner ear complaints. Cervical proprioception [15] and posture are integral parts of multisensory integration responsible for balance and orientation. Neck injury can lead to alterations in proprioception, joint alignment, and possible disruption of vascular supply to the brainstem [16]. Neck injury can lead to a multitude of debilitating symptoms including vertigo and dizziness, which are common and enigmatic [17] problems.

Based on data about working conditions and exposure to free-time noise, significant numbers of people in the EU are exposed to the damaging effects of noise, and about 13% have communication problems that stem almost exclusively from being hard of hearing [18]. Among noise-exposed workers, 25% are exposed to impulse noise [19]. In humans, noise may cause inner ear trauma and affect endolymph circulation [20]. Sound-induced vestibular derangement has been recorded in animal studies and found to cause secondary endolymphatic hydrops [21,22]. In individuals with acute acoustic trauma, Wang et al. [23] saw abnormalities in the saccular function measured with a cVEMP test. However, no patient complained of vertigo in that study, suggesting that the abnormal cVEMP was not associated with MD. Even chronic noise exposure has led to significant reduction in vestibular function in humans, as shown in cVEMP and oVEMP tests [24]. The bulging of Reissner’s membrane has been seen in animals after infrasound exposure, indicating a hydrops formation [21] or post-traumatic endolymphatic hydrops. However, the role of environmental noise in the comorbidity or etiology of MD in humans has been underexplored [12].

The aim of the present study was to evaluate the complaint profiles of MD patients with special reference to comorbidities and possible contributing factors such as TBI, neck injury, and acoustic trauma. We believe that by examining these associations in MD, we may understand if the occurrence of these external factors induces and/or modifies specific complaint patterns of MD patients.

## 2. Methods

### 2.1. Study Design

The researchers in this study used a retrospective design to analyze anonymous register data from the Finnish Ménière Federation (FMF). The registry contained detailed data the FMF had collected from their members when establishing a computer-based diagnostic and peer support program during the years 1998–2016 [25]. Under Finnish law, anonymous registry data collected by a patient association does not require ethical approval, although the researchers did obtain permission from the FMF to analyze its data for the purpose of this study.

### 2.2. Participants

Data were collected from 961 of the original 1200 FMF members; it should be noted that that number has since increased to 1646 members. From the existing data, information was missing in 50 subjects; therefore, 912 patients were included in the current study. Each was mailed a 26-page questionnaire with a stamped, addressed envelope for the return of their responses. The items in the questionnaire have been used in the researchers’ previous studies on MD [26,27]. If a participant did not reply, they were reminded up to three times. If information was missing in their responses, the subjects were contacted by phone, and the data was fed into the database.

The mean age of the participants was 60.2 years (range 25 to 80 years, SD = 12.1 years). The mean duration of the disease was 12.6 years (range 0.5 to 50 years, SD = 11.2 years). Participants included 717 females (i.e., 78.7%) and 194 males (i.e., 21.3%) corresponding to the gender distribution of the FMF and the prevalence of the willingness to reply to questions in Finland [28]. MD was diagnosed in these participants via a computerized inference engine and based on symptoms defined in the diagnostic criteria of the American Academy of Otolaryngology–Head and Neck Surgery (AAO-HNS) [29].

### 2.3. Data Collection

Descriptions of vestibular complaints are often difficult to classify correctly due to linguistical variability, teaching habits, complaint characterization, and clinical practice [30]. In the Finnish language, for example, there is no definitive distinction between *vertigo* and *dizziness*. In this paper, we attempt to characterize types of vestibular complaints based on the timing of the complaint, what triggers the dizziness, and possible associated or triggering factors. The instrument used to collect data was an 86-item otoneurology questionnaire containing disease-specific and impact-related questions [31]. General health-related quality of life was evaluated using the EQ-5D-3L questionnaire [2]. The E-QoL (i.e., EQ-5D-3L; *(E-QoL-5d instrument: https://euroqol.org/eq-5d-instruments (accessed on 9 January 2022)) generic health measure consists of five questions at three levels and a visual analog (VAS) scale. In general, the complaint-specific interference was assessed by asking the subjects to rate severity or frequency of their complaints and their impacts on a five-point scale ranging from “none” to “very severe”. Vertigo attacks, balance problems, and vestibular drop attacks (VDA) were assessed by character, provoking items, frequency, severity, and how much impact they caused. In addition, the impacts of tinnitus, hearing loss, and hyperacusis were also gathered. All case histories were self-reported (see Appendix A) [31].

Injury to the inner ear was assessed with four separate questions. First, we asked a direct question about injury to the head or neck associated with the onset of complaints that had occurred within six months of the event. To assess head trauma, the classification of TBI based on self-reported duration of transient loss of consciousness (TLoC) was used. The responses were classified into three categories: (a) mild TBI with no TLoC, (b) moderate TBI with TLoC that lasted less than two hours, and (c) severe TBI (such as brain contusions) when TLoC lasted more than two hours [6]. Finally, reports of exposure to occupational noise exceeding an 85 dB (A) noise level for longer than five years were also recorded. According to the European Noise Directive, a sound pressure level of 80 dB (A) or more can cause permanent hearing loss. These key questions are provided in Appendix A.

### 2.4. Data Analyses

For categorical data as well as variables without normal distribution, non-parametric tests including the Mann–Whitney U test, Chi square, Kendal’s Tau correlations, and Kruskal–Wallis H test were used to investigate the association of complaints with MD. For continuous variables with normal distribution, Student’s *t*-test and ANOVA were used to evaluate group differences. Bonferroni post hoc tests were performed as secondary analysis where necessary. A *p*-value of 0.05 was used for interpretation of statistical significance.

## 3. Results

### 3.1. Traumatic Brain Injury

Of the 912 participants, 175 subjects reported a TBI (19.2%). When compared to reference cases without TBI, the TBI-group experienced VDA, head movement-induced vertigo, presyncope, physical strain-induced vertigo, problems rising from a chair, reduced E-QoL, and headache with vertigo (Table 1).

**Figure 1:**
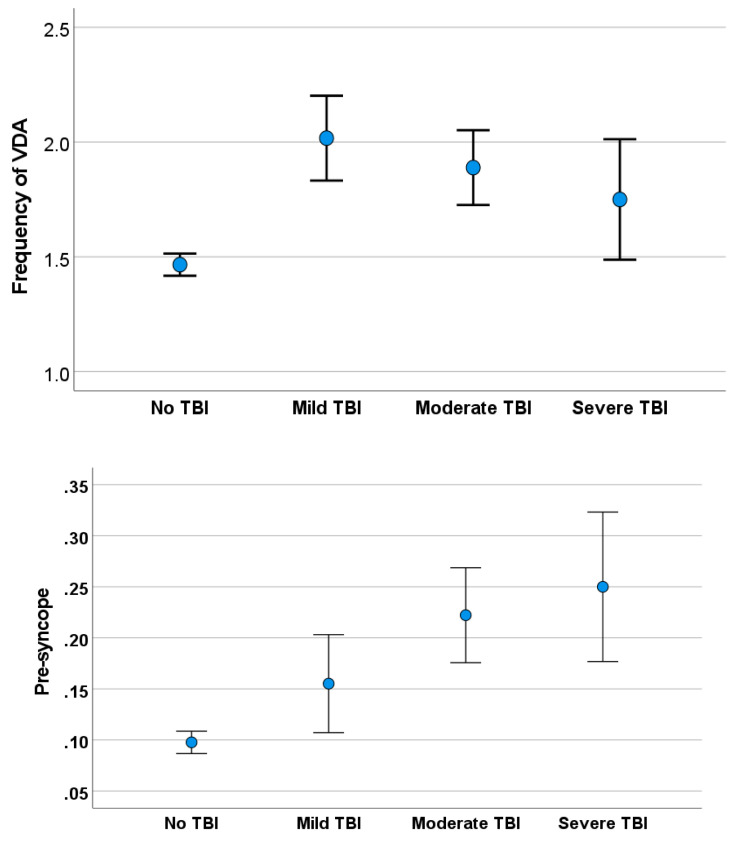
Frequency of vestibular drop attacks (VDA) in subjects with TBI and in the control group (**upper**) and of presyncope in subjects with TBI and the control group (**lower**).

TBI was classified as mild in 58 participants for whom there was no TLOC; as moderate in 81 participants, with TLOC lasting less than two hours; and as severe in 36 participants, with TLOC lasting more than two hours. When exploring the association of severity of TBI with complaints, we observed significant differences with VDA (χ^2^ = 15.29, *p* < 0.001), presyncope (χ^2^ = 15.81, *p* < 0.001) (see Figure 1), and physical strain-associated vertigo (χ^2^ = 9.03, *p* = 0.003).

**Figure 2:**
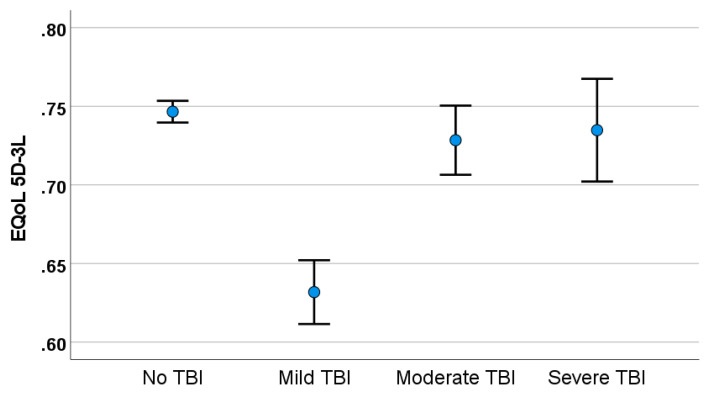
Health-related quality of life (E-QoL-5D-3L) in MD and severity of TBI.

Logistic regression was performed to examine whether any of the complaints predicted impact on MD patients in different TBI groups (see Table 2). Of the four separate models, the complaints explained only 2–6% of the TBI in different groups with VDA, headache associated with vertigo, E-QoL (see Figure 2), and mobility problems being the significant predictors in different models.

### 3.2. Neck Trauma Associated with Vertigo

Neck trauma, mostly whiplash-type, was reported by 99 patients (10.8%). However, only 47 (5.2%) reported neck trauma associated with vertigo (NTwV). The NTwV group experienced VDA, postural imbalance, problems rising from a chair, the impact of hyperacusis, and fatigue more than the reference group without NTwV (see Table 1). The majority of the participants (85%) with NTwV had TBI. Of the 47 participants with NTwV, 14 had mild TBI, 22 had moderate TBI, and 4 had severe TBI.

**Figure 3:**
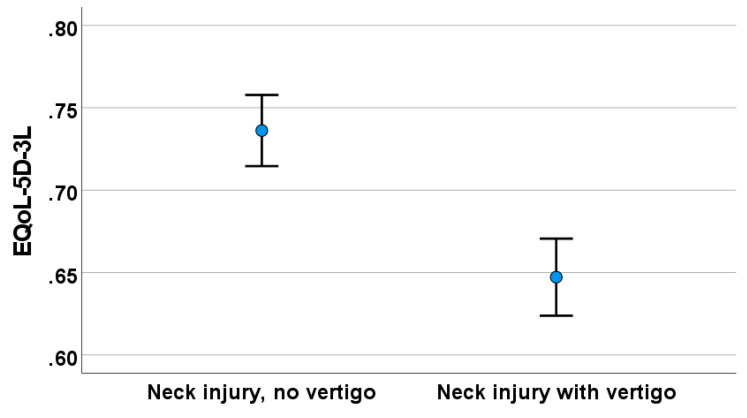
Health-related quality of life (E-QoL-5D-3L) among study participants with and without NTwV.

In a detailed analysis of the EQ-5D-3L data, the NTwV group had poor E-QoL (i.e., 68%) compared to those in the neck injury without vertigo group (i.e., 74%), as illustrated in Figure 3. These differences were statistically significant (*t* = 2.28, *p* = 0.007).

In logistic regression analysis, the significant complaints associated with NTwV were motility problems, which explained 9.8% of the variability of NTwV.

### 3.3. Noise-Induced Hearing Loss

Chronic occupation noise exposure over 85 dB (A) for longer than five years was reported by 230 participants (i.e., 25.2%). None of the vertigo associated variables (i.e., character of vertigo, frequency of attacks, severity of attacks, nausea) or balance and gait problems (i.e., unsteadiness frequency, unsteadiness severity, and gait problems) differed between the groups with the noise-exposed and reference groups. However, the noise-exposed group experienced more impact of hearing loss, impact of hyperacusis, anxiety, and headache with vertigo than the reference group without chronic noise exposure (see Table 1).

**Figure 4:**
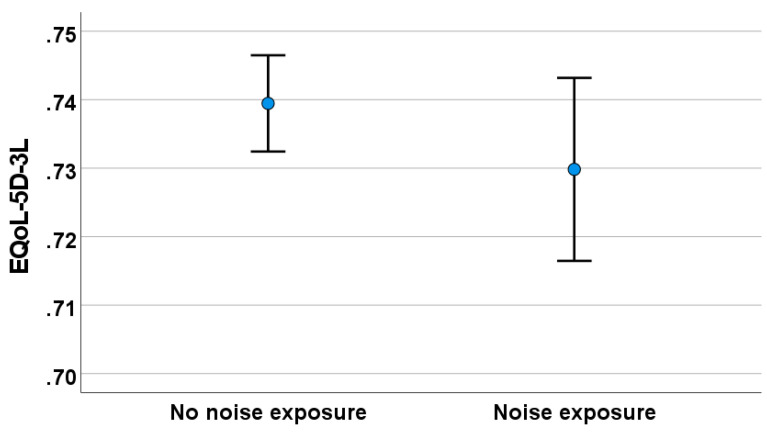
Health-related quality of life (E-QoL-5D-3L) among study participants with and without noise exposure.

No difference (*t* = 0.118, *p* = 0.080) was observed in the E-QoL between the noise-exposed and reference groups (see Figure 4).

Logistic regression was performed to examine whether any of the complaints predicted the impact on MD patients with chronic noise exposure. The model was statistically significant and explained 4.8% of the variability. VDA, headache-associated vertigo, severity of tinnitus, and impact of hearing loss were variables that significantly contributed to the model prediction.

## 4. Discussion

The aim of the current study was to examine the comorbidity of TBI, NTwV, and noise trauma interference with the phenotype of complaints in MD. Various associations were noted between the groups with and without TBI, NTwV, and noise exposure, although many associations were weak and only marginally statistically significant (see Table 1). Logistic regression models suggest that the complaints explain only 2–10% of the variability in TBI, NTwV, and noise exposure (see Table 2), highlighting the heterogeneity of MD. One reason for noting the weak models is also to acknowledge factors that were important but not included in this study, such as biological susceptibility, hereditability, thyroid function, and migraine [27,32]. We observed that TBI and NTwV comorbidity with MD were associated with balance and gait problems, indicating vestibular damage. However, chronic noise exposure seems to worsen complaints related to hearing loss in MD.

### 4.1. Effect of Traumatic Brain Injury

TBI is an enormous public health concern. It can be defined as mild, moderate, or severe, and it is often classified in the literature based on duration of TLoC or on the Glasgow coma scale [33]. However, such classifications may be misleading, as mild TBI can lead to long-term disability, and severe injury can sometimes be more manageable [5]. We observed that the E-QoL was significantly reduced after mild TBI; this supports the idea that the severity of TBI classified by the duration of the TLoC does not mirror the impact on the E-QoL. Among the most common complaints after mild TBI are headaches, dizziness, fatigue, and cognitive difficulties, with about 13% of patients unable to continue to work [5,8]. The variability in the incidence rate may depend on the definitions of the severity of TBI and by the time elapsed since the injury [34]. The incidence of dizziness, even with mild TBI, is common and ranges from 17% to 72% [8,34,35]. In one of the few long-term studies on untreated patients with mild TBI, Berman and Frederickson [9] showed vertigo persisting in 59% of patients after five years of recovery. Integrating cognitive impairment, consciousness duration, and imaging results may be useful in the assessment of the severity of TBI [6] and be more accurate than basing it on the duration of TLoC. Due to factors like post-traumatic amnesia, the duration of TLoC can be misleading if relevant eyewitnesses are not present.

Researchers [3,10,36,37] have investigated whether TBI can cause MD or change related complaint patterns. Marzo et al. [5] reported the main complaint after mild TBI was disequilibrium with tinnitus and headache; and two out of their 16 subjects developed MD during follow-up stages. In a different, cross-sectional study among 4291 head-injured workers, the most common peripheral vestibular disorder identified was positional vertigo [8]. A significant group was defined as non-defined otolith disorder. A few were classified as delayed endolymphatic hydrops, and some fulfilled the diagnostic criteria for MD. In addition, some individuals had a history of VDA, episodic rocking, or translational pulsion. Our results support the idea that the main impact of TBI on MD was related to the malfunctioning of the otolith system. We found no association with the cardinal symptoms of MD: vertigo, ear fullness, and hearing loss. Thus, the results do not support the idea that TBI would be a causative factor for MD in provoking endolymphatic hydrops. Logistic regression analysis showed the comorbidity of TBI with MD explained only 2–6% of the variability in complaints.

Although syncope is rare in MD, we have found [11] that about 5% of MD patients will have syncope in VDA and suggested the response is mediated by the sympathetic vestibular reflex. Ayabe et al. [38] showed that a head-up tilt test is the gold standard for assessing neutrally mediated syncope, supporting our findings that the effect of TBI is associated with otolith function.

The findings of this study showed that in TBI correlated with VDA, balance and gait problems related to derangements of the vestibular system significantly reduce the E-QOL. Motility problems were observed among severe TBI as a comorbidity in MD, indicating that such joint disability should be noted for rehabilitation purposes, as has been previously indicated [39].

In studies evaluating otolith organ dysfunction after mTBI and blast injury, abnormal cVEMPs were recorded in abundant amounts: 52% by Akin and Murnane [40], 52% by Ernst et al. [39], and 29% by Lee et al. [4]. Another study [41] found abnormalities occurred more frequently for measures of cVEMP (25%) and oVEMP (18%) than for measures of horizontal semicircular canal function (8% for the slow harmonic acceleration test and 6% for the caloric test). From this data [41], we can safely conclude that TBI and blast injury cause major effects on the otolith system. Further clinical data on postural derangement and the frequent association with BBPV also suggested injury-associated complaints were characterized by otolith damage. Balance problems occurred in 72% of individuals having chronic dizziness after mTBI and blast injury that was more frequent than abnormalities in 34% of the vestibular test [41]. Due to the anatomic proximity of the saccule to the stapes footplate, it may be particularly susceptible to trauma and blast noise-induced damage [41]. Thus, complaints of balance function suggest balance-associated complaints in MD may be caused more frequently by otolith damage than semicircular canal damage. Ernst et al. [39] concluded that besides otolith damage, they observed many other inner ear problems, such as delayed endolymphatic hydrops. Although the pathological finding of an inner ear response in MD is endolymphatic hydrops, we found no evidence in this study that either TBI or NTwB would aggravate rotatory vertigo spells. Current imaging methods provide the potential to further study the association of endolymphatic hydrops in mTBI in more detail and should be carried out [41]. In a previous study endolymphatic hydrops has been found in various inner er disorders such as tinnitus, sudden deafness, and vestibular neurotis, among others, indicating that endolymphatic hydrops may be a sign similar to the elevation in the sedimentation rate in infection; reacting nonspecifically to the incidence [42].

### 4.2. Effect of Neck Trauma with Vertigo

Ever since the experiments of Magnus [43], who showed that the tonic neck reflex arose from receptors supplied by the upper cervical segments, the neck has been regarded as an important organ in postural processes, including gait. Disturbances in gait may be, in fact, produced in experimental animals either by damaging or anesthetizing neck muscles [15] or by cutting upper dorsal roots [44]. In humans, dizziness and ataxia may be expected to follow damage or anesthesia of the neck muscles or whiplash injuries (for review, see [17]). Neck injury in connection with TBI or in whiplash-type disorders has been found to cause cervical vertigo in humans, although conclusive evidence is lacking. Vast evidence indicates that neck problems impair postural control [45] and cause presyncope [17]. Yet, the receptors participating in cervicocollic reflexes, or any other postural reflexes, have not been fully identified. It is nevertheless doubtful whether information from the large neck muscles contributes to the tonic neck reflexes, since McCough et al. [46] showed that tonic neck reflexes are not lost following section or denervation of the main neck muscle, whereas cutting off the nerves innervating tissue close to the intervertebral joints abolishes these reflexes.

A large congregation of muscle spindles is present in the short muscles around the perivertebral tissue of both humans and cats [47], and a deprivation of the afferent input from these receptors is known to cause postural deficits [13]. Golgi tendon organs (GTO) were also observed in the same regions as muscle spindles in dyads with spindles. The receptors are strategically located in the vicinity of joints in a position where they can monitor changes in the length or tension of the neck muscles. A neck injury that has not been recorded in the present study is one that may arise from injury of the perivertebral joints.

The results of the present study indicate that the participants with NTwV had complaints that could be associated with vestibular damage. So far, the association of neck trauma with vestibular system ailments has not gained scientific acceptance, as indicated in a recent Barany Society position paper [48]. However, the observed vestibular problems, which were postural imbalance, problems rising from a chair, the impact of hyperacusis, fatigue, and a reduced E-QoL, could be due to head injury, as a majority of the victims with NTwV had simultaneous TBI.

### 4.3. Effect of Noise Trauma

We did not observe that occupational noise exposure would influence vestibular problems in MD, so we find individual susceptibility to hearing loss may not be interactive with mechanisms leading to MD. To demonstrate risk prediction in the ISO hearing loss database [49], a model for noise-induced hearing loss with an exposure of 100 dB (A) for 8 h a day over 30 years gave a median noise-induced hearing loss of 45 dB with a variation of 60 dB (10th–90th percentiles) at 4 kHz. Thus, depending on susceptibility to noise, subjects may have normal hearing or profound hearing loss.

The EU directive [50] sets improved limits to allowable noise levels and new requirements to control individual susceptibility factors, but the directive does not define these factors. This may be biased by inaccuracies in noise exposure evaluation or by age-related hearing loss and the interference of other ear diseases [51,52]. We did not measure the hearing level but used subjective impact evaluation. Although a deeper analysis of noise effects on the vestibular system is needed, the present study contributes to a road map in that it shows chronic noise exposure does not aggravate MD.

Impulse noise damages the inner ear, producing up to 10 dB greater hearing loss than steady state noise, but in industry, the mandatory use of hearing protectors can restrict the damaging effects of impulse noise [53]. In TBI, the hitting of the skull also produces, through high deceleration, impulse noise and may partly explain the vestibular complaints in the present study.

Although in the present study, 26.1% of participants had probable bilateral MD and reported hearing loss in both ears, we have observed, in another study, up to 70% bilateral endolymphatic hydrops in the MRIs of MD patients, and despite having bilateral endolymphatic hydrops, most of those patients considered these “healthy ears” [54]. The subjects with bilateral hearing loss had, in the present study, a more profound impact on hearing than those with unilateral hearing loss. Those with severe TBI had more hearing problems than those with no TBI or mild or moderate TBI. In mild or moderate TBI and in NTwV with bilateral MD, there was no difference in subjective hearing impact. The same was also true for the vertigo and balance variables, indicating that bilateral MD may not behave differently from unilateral MD after TBI and NTwV with the exception of VDA.

Studies using the guinea pig model for short- and long-term noise exposure have shown the saccule can exhibit temporary/permanent functional loss resembling hearing threshold shifts [34]. Secondary hydrops might also have a different clinical phenotype, although in terms of tinnitus, ear fullness, nausea/vomiting, and vertigo/dizziness, that phenotype has not been shown [21]. In line with our results, Segal et al. [12] could not confirm that MD would be caused by noise exposure. Chronic noise damage of the cochlea has been related to metabolic stress, leading to the production of reactive oxygen species, reactive nitrogen species, and other free radical molecules in the cochlea [35]. We find, therefore, that chronic noise can create hearing problems and tinnitus in MD.

### 4.4. Study Limitations

This study has a linguistic limitation. In the United States, for teaching purposes, vestibular problems are classified in standard categories of vertigo, presyncope, disequilibrium, and nonspecific dizziness. However, patients cannot always classify their symptoms into a category, or they fit into two categories [30]. Descriptions of the quality of dizziness are unclear, inconsistent, and unreliable, casting doubt on the validity of the traditional approach to the patient with dizziness [55]. In Finnish, the word for “vertigo” indicates not only vertigo, but also dizziness, balance problems, and others. Therefore, in the present paper, we attempted to characterize the type of vestibular complaint with the timing of the complaint and what triggers the dizziness. We found that *vertigo* and *dizziness* can be misinterpreted as balance and gait problems and light-headedness. The misunderstanding of patient-oriented terminology may bias the outcome of the results, and all complaints might be covered under the umbrella of dizziness. The current study had a higher percentage of female participants. This is dependent on the FMF population and the response rate of female participants.

As the current study is based on self-reports, there are several related limitations. For instance, recall bias may lead to the inaccurate reporting of the history of TBI and actual duration of unconsciousness. Moreover, as the study participants were anonymous, we could not retrieve the retrospective hospital reports and study the trauma mechanisms and duration of TLoC in detail. Further, posttraumatic amnesia may interfere with patient reporting and the classification of mild TBI versus moderate TBI. We also related the onset of complaints within 6 months from the TBI. Therefore, the triggering of MD by TBI is difficult to confirm. Although the most common reason for neck trauma was a whiplash-type injury, there are no objective tests or reliable histories to classify the etiology of neck trauma. For these reasons, the results of the current study should be interpreted with caution and should be treated as exploratory.

We did not perform any vestibular tests such as oVEMP or cVEMP to confirm the involvement of otolith dysfunction. Therefore, the specific involvement of otoliths as the reason for vertigo after TBI and NTwV is only suggestive as trauma to the semicircular canals is also likely to occur.

## 5. Conclusions

We evaluated whether TBI, NTwV, or occupational noise exposure as comorbidities in MD could cause variability in the phenotype of MD. Independent from the character and severity of TBI or NTwV, the complaint patterns suggested otolithic dysfunction. It is important to note that even mild TBI may result in long-term consequences. This is in line with our observation that the E-QoL was significantly worsened after mild TBI. Further, TBI and NTwV explained the variability of complaints by 2 and 10%, respectively. Moreover, occupational noise exposure seemed to impact hearing loss and tinnitus but not vertigo.

## Figures and Tables

**Table 1:** Complaint-related phenotype differences in participants with TBI, neck injury, and chronic noise exposure compared to reference groups without these conditions. Statistically significant findings are marked with stars (* *p* < 0.05, ** *p* < 0.01). Complaints are classified by time or impact scale ranging from 0–5, where 0 is no and 5 indicates very severe impact (see Appendix A). Note *: In the NTwV group and in the reference group, only cases without TBI are included. The NTwV group had vertigo associated with neck trauma.

	TBI Analysis		Neck Injury Analysis		Chronic Noise Exposure Analysis	
Complaints	Reference Group (n = 737) Mean	TBI Group (n = 175) Mean	Reference Group (n = 52) Mean	NTwV Group (n = 47) Mean	Reference Group (n = 682) Mean	Noise Exposure Group (n = 230) Mean
Vertigo frequency	1.99	2.02	2.06	2.24	2.09	2.03
Vertigo spell duration	2.80	3.01	2.85	2.88	2.87	2.79
Vertigo spell intensity	3.13	3.19	3.04	3.02	3.15	3.19
Severity of nausea	2.32	2.52	2.45	1.81	2.40	2.28
Frequency of VDA	1.46	1.90 **	1.40	2.31 *	1.59	1.45
Head movement indued vertigo	1.39	1.67 **	1.43	1.89	1.47	1.40
Pre-syncope	0.10	0.21 **	0.09	0.23 *	0.19	0.26
Physical strain induced vertigo	1.01	1.24 *	1.04	1.55 *	1.05	1.09
Postural imbalance	1.38	1.51 **	1.46	1.86 *	1.43	1.32
Gait problems	0.48	0.61	0.45	0.76	0.51	0.50
Problems with arising from chair	0.42	0.50 *	0.32	0.63 *	0.41	0.50
Impact of hearing loss	1.29	1.33	1.30	1.39	1.25	1.44 **
Impact of tinnitus	1.62	1.88	1.56	1.73	1.57	1.81 *
Impact of hyperacusis	1.67	1.33	1.87	2.10 *	1.62	1.88 **
E-QoL VAS scale	72.33	69.75	72.5	67.2	72.0	71.4
E-QoL EQ-5D-3L	0.75	0.70 **	0.74	0.65 **	0.74	0.73
Anxiety	1.43	1.57	1.32	1.67	1.42	1.57 *
Fatigue	1.22	1.32	1.15	1.58 **	1.22	1.28
Headache with vertigo	0.72	0.95 *	0.72	1.04	0.7	0.92 *

**Table 2:** Outcome of logistic regression analysis on factors predicting outcome for different TBI, NTwV, and chronic noise exposure groups.

	Any TBI (n = 175)	Mild TBI (n = 58)	Moderate TBI (n = 81)	Severe TBI (n = 36)
Negelkerge sq	0.034	0.064	0.018	0.018
Chi Square test and significance	Χ^2^ = 19.4, *p* < 0.001	Χ^2^ = 21.1, *p* < 0.001	Χ^2^ = 7.2, *p* = 0.007	Χ^2^ = 4.9, *p* = 0.027
Complaint 1	VDA	Poor E-QoL	VDA	Motility problems
Complaint 2	Headache associated with vertigo	VDA		

## Data Availability

The original contributions presented in the study are included in the article, further inquiries can be directed to the corresponding author.

## Appendix A. Questions Related to Head Injury, Neck Injury and to Exposure to Environmental Noise

1. Do you have a direct injury to the head or neck which was associated with the onset of vertigo symptoms (which occurred within 6 months of the event)?
2. Did you experience unconsciousness lasting less than 2 h with your head injury? In what year did this happen?
3. Did you experience any whiplash-type injury to the neck? In what year did this happen?
4. Did you experience any direct injury of the ear, acute noise injury, or bleeding from the ear which would have caused hearing loss or tinnitus? In what year did this happen?
5. Were you exposed to loud noise at work (a noise level exceeding 85 dB (A)) for more than 5 years?
